# Pragmatic nationwide master observational trial based on genomic alterations in advanced solid tumors: KOrean Precision Medicine Networking Group Study of MOlecular profiling guided therapy based on genomic alterations in advanced Solid tumors (KOSMOS)-II study protocol KCSG AL-22–09

**DOI:** 10.1186/s12885-024-12338-y

**Published:** 2024-05-09

**Authors:** Sun Young Kim, Jee Hyun Kim, Tae-Yong Kim, Sook Ryun Park, Shinkyo Yoon, Soohyeon Lee, Se-Hoon Lee, Tae Min Kim, Sae-Won Han, Hye Ryun Kim, Hongseok Yun, Sejoon Lee, Jihun Kim, Yoon-La Choi, Kui Son Choi, Heejung Chae, Hyewon Ryu, Gyeong-Won Lee, Dae Young Zang, Joong Bae Ahn

**Affiliations:** 1 Department of Oncology, University of Ulsan College of Medicine, Asan Medical Center, Seoul, South Korea; 2 Department of Internal Medicine, Seoul National University College of Medicine, Seoul National University Bundang Hospital, Seongnam, South Korea; 3 Department of Internal Medicine, Seoul National University College of Medicine, Seoul National University Hospital, Seoul, South Korea; 4 Department of Internal Medicine, Korea University College of Medicine, Korea University Anam Hospital, Seoul, South Korea; 5 Department of Internal Medicine, Samsung Medical Center, Sungkyunkwan University School of Medicine, Seoul, South Korea; 6 Division of Medical Oncology, Department of Internal Medicine, Yonsei University College of Medicine, Yonsei Cancer Center, Seoul, South Korea; 7 Center for Genomic Medicine, Seoul National University Hospital, Seoul, South Korea; 8 Center for Precision Medicine, Seoul National University Bundang Hospital, Seongnam, South Korea; 9 Department of Pathology, University of Ulsan College of Medicine, Asan Medical Center, Seoul, South Korea; 10 Department of Pathology and Translational Genomics, Samsung Medical Center, Sungkyunkwan University School of Medicine, Seoul, South Korea; 11 Department of Cancer Control and Population Health, Graduate School of Cancer Science and Policy, National Cancer Center, Goyang, South Korea; 12 Department of Internal Medicine, National Cancer Center, Goyang, South Korea; 13 Division of Hematology and Oncology, Department of Internal Medicine, Chungnam National University Hospital, Chungnam National University College of Medicine, Daejeon, South Korea; 14 Division of Hematology-Oncology, Department of Internal Medicine, Institute of Health Science, Gyeongsang National University Hospital, Gyeongsang National University College of Medicine, Jinju, South Korea; 15 Department of Internal Medicine, Hallym University College of Medicine, Hallym University Sacred Heart Hospital, Anyang, South Korea

**Keywords:** Master observational trial, Molecular tumor board, Next-generation sequencing

## Abstract

**Background:**

Next-generation sequencing (NGS) has been introduced to many Korean institutions to support molecular diagnostics in cancer since 2017, when it became eligible for reimbursement by the National Health Insurance Service. However, the uptake of molecularly guided treatment (MGT) based on NGS results has been limited because of stringent regulations regarding prescriptions outside of approved indications, a lack of clinical trial opportunities, and limited access to molecular tumor boards (MTB) at most institutions. The KOSMOS-II study was designed to demonstrate the feasibility and effectiveness of MGT, informed by MTBs, using a nationwide precision medicine platform.

**Methods:**

The KOSMOS-II trial is a large-scale nationwide master observational study. It involves a framework for screening patients with metastatic solid tumors for actionable genetic alterations based on local NGS testing. It recommends MGT through a remote and centralized MTB meeting held biweekly. MGT can include one of the following options: Tier 1, the therapeutic use of investigational drugs targeting genetic alterations such as *ALK, EGFR, ERBB2, BRAF, FH, ROS1,* and *RET*, or those with high tumor mutational burden; Tier 2, comprising drugs with approved indications or those permitted for treatment outside of the indications approved by the Health Insurance Review and Assessment Service of Korea; Tier 3, involving clinical trials matching the genetic alterations recommended by the MTB. Given the anticipated proportion of patients receiving MGT in the range of 50% ± 3.25%, this study aims to enroll 1,000 patients. Patients must have progressed to one or more lines of therapy and undergone NGS before enrollment.

**Discussion:**

This pragmatic master protocol provides a mass-screening platform for rare genetic alterations and high-quality real-world data. Collateral clinical trials, translational studies, and clinico-genomic databases will contribute to generating evidence for drug repositioning and the development of new biomarkers.

**Trial registration:**

NCT05525858.

## Background

The rapid development of molecularly targeted agents and immunotherapies, coupled with high-throughput tumor profiling using next-generation sequencing (NGS), is steering medical oncology towards “precision medicine.” Several precision medicine clinical trials in the United States and some European countries have adopted pragmatic platform trial designs [1–5]. Some studies have demonstrated that molecular profiling-guided therapy (MGT), determined by the genomic profile of a tumor and assessed by a molecular tumor board (MTB), may improve clinical outcomes in patients with refractory solid tumors [2, 6].

However, challenges remain with the clinical implementation of MGTs [7]. Molecular profiling remains expensive and is very intricate in terms of the number of target genes, variant calling procedures, and sequencing techniques [8]. Meanwhile, the expertise in interpreting and matching MGTs often varies among oncologists, especially those who work at community hospitals. Regulatory approval for MGTs is limited to very narrow indications (e.g., trastuzumab is approved for *HER2*-amplified breast or gastric cancer but not for *HER2*-amplified biliary or salivary gland cancer), and it is difficult to prescribe MGTs outside of regulatory approval for patients with rare actionable genomic alterations. In Korea, where prescriptions outside the approved indications are strictly controlled, access to MGT is limited unless patients participate in clinical trials using MGTs. However, the opportunity to enroll in clinical trials is restricted in Korea, especially outside the Seoul Metropolitan area [9].

We have previously conducted a pragmatic precision medicine trial, the KOSMOS-I pilot study, designed as a nationwide, prospective, multicenter, multi-cohort study of MGT within local clinical practice. Local NGS reports from patients with refractory metastatic solid tumors were assessed by a central molecular tumor board (cMTB), which convened twice weekly on a virtual platform. MGT options were provided in the form of the Therapeutic Use of Investigational Drugs (TUID) program, approved for individual patients by the Ministry of Food and Drug Safety (MFDS). MGT was found to be feasible for 51.3% (99/193) of the patients enrolled over just one year, from February 2021 to February 2022. This finding underscores the significant need for MGT in Korea and demonstrates the feasibility of MGT supported by nationwide cMTB [10].

Based on these results, we expanded the KOSMOS-I platform to enroll a larger number of patients. We designed a master observational trial (MOT), KOSMOS-II, which offers more MGT options, including TUID and investigator-initiated clinical trials (IITs), as part of the entire MOT framework. In this context, we describe the rationale and design of the KOSMOS-II trial, the current progress of this project, and discuss the operational issues and perspectives concerning this pragmatic platform.

## Methods/design

### Study design

**Fig. 1:**
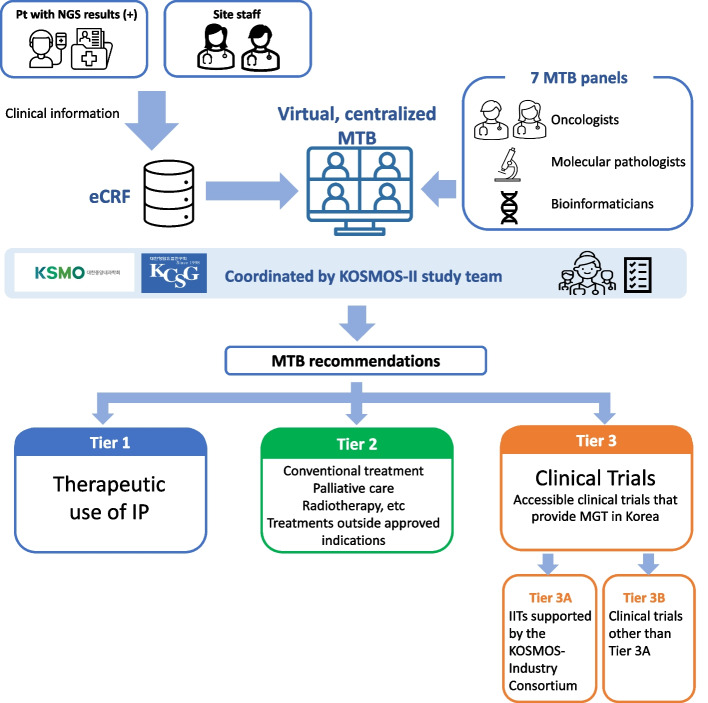
Study Scheme for KOSMOS-II trial. Abbreviations: NGS, next-generation sequencing; eCRF, electronic case report form; MTB, molecular tumor board; KSMO, Korean Society of Medical Oncology; KCSG, Korean Cancer Study Group; IP, investigational products; MGT, molecular profile guided therapy; IIT, investigator initiated clinical trials; Pt, patient

The KOSMOS-II trial comprises a framework for screening patients with actionable targets, operating a cMTB that recommends and provides MGT, and developing a clinico-genomic database (CGDB). MGT options include TUID with targeted and/or immunotherapies (Tier 1); local practice involving therapy within or outside the approved indications (Tier 2); or participation in clinical trials (Tier 3) (Fig. 1).

### Study objectives

The primary objective of this study is to evaluate the feasibility of MGT in terms of the proportion of participants who received the treatment (MGT rate). The receipt of MGT is defined as receiving at least one dose of MGT targeting the genetic alterations (GAs) detected by NGS, regardless of the tier assigned by the cMTB. The second primary objective is to evaluate the effectiveness of MGT in terms of clinical benefit rate (CBR: the percentage of patients with complete response, partial response, or stable disease according to Response Evaluation Criteria in Solid Tumors (RECIST) 1.1 [11] beyond 16 ± 2 weeks of treatment) in Tier 1.

The secondary objectives include the following:

1. To evaluate the effectiveness of MGT in terms of the objective response rate, progression-free survival, treatment duration, and 1-year overall survival in Tier 1 and Tier 3A (investigator-initiated trials by KOSMOS-II study team), where the clinical outcomes of MGT are being captured.
2. To evaluate the safety of MGT according to Common Terminology Criteria for Adverse Events (CTCAE) version 5.0
3. To assess the operational feasibility of delivering MGT.
4. To correlate the molecular profile with the clinical outcomes of MGT.

In this study, the explorative objectives are divided into translational and clinical objectives. The translational objectives are presented in the following section. The clinical explorative objective is to evaluate the extension of the MGT beyond the KOSMOS-I. In the KOSMOS-I trial, MGT was provided for up to 12 months. Therefore, the KOSMOS-II trial will enroll the patients after the completion of study treatment of KOSMOS-I if they do not experience disease progression to provide extended MGT. The clinical and molecular characteristics of the patients (prior KOSMOS-I participants) will be described separately from the whole study population.

### Eligibility criteria

Patients with locally advanced or metastatic solid tumors are eligible for the study based on the following criteria: (1) progression on standard treatments or exhaustion of available treatment options; (2) patients have an available NGS report for the tumors, ideally obtained within the last 3 years, and provided by laboratories accredited by MFDS or regulatory bodies compatible with MFDS, such as Clinical Laboratory Improvement Amendments; (3) a life expectancy of at least 12 weeks; (4) observable adequate recovery from the most recent systemic or local treatment.

### Study treatment

Table 1 presents the options available for Tier 1. These options are provided through TUID, which is available only for drugs that have already been approved for certain indications by MFDS, and it does not include investigational products without any approved indications. The KOSMOS-II centralized and standardized the TUID approval process for study participants. The protocol specifies the actionable GAs that match each drug on the Tier 1 list; however, the cMTB panel can recommend Tier 1 options if there is evidence that GAs other than the pre-specified ones can predict the benefit from the drug.

**Table 1:** Tier 1 options available through therapeutic use of investigational drugs program

Cohort	Drugs	Drug-specific genetic alterations^a^
Cohort A	Alectinib	*ALK* fusion
Cohort B	Atezolizumab	MSI-H
Cohort C	Erlotinib	*EGFR* mutation
Cohort D	Trastuzumab + Pertuzumab	*ERBB2* amplification or overexpression
Cohort E	Trastuzumab emtansine	*ERBB2* amplification, overexpression, or mutation
Cohort F	Vemurafenib	*BRAF* V600E/D/K/R mutations
Cohort G	Bevacizumab + Erlotinib	*FH* inactivating mutations
Cohort H	Entrectinib	*ROS1* gene fusion
Cohort I	Pralsetinib	*RET* fusion

^a^The condition which the MTB panels recommend the Tier 1 options is not limited to these pre-specified list of genetic alterations and the MTB panel can provide Tier 1 options based on the evidence level determined according to Korean Precision Medicine Networking Group scale of clinical actionability of molecular target (K-CAT)

Tier 2 options include treatment according to local practice, such as chemotherapy for approved indications, if any, treatment outside approved indications that has been allowed by the Health Insurance Review and Assessment Service (HIRA), or best supportive care.

Tier 3 options, which involve participation in clinical trials, are always prioritized over Tier 1 or Tier 2 options, provided they are available. Clinical trials that assess MGT and are accessible in Korea are recommended. In particular, the opportunity to participate in IITs, supported by the KOSMOS-industry consortium (Table 2), is recommended if the patient is involved in the KOSMOS-II trial and the cMTB panel determines that the GAs from the patient’s tumor match the eligibility criteria of the IITs. These IITs, referred to as Tier 3A options, are designed in alignment with the master protocol of the KOSMOS-II trial to expedite the screening procedure within the KOSMOS-II platform and offer additional MGT options for KOSMOS-II participants while allowing them to explore the possibility of repurposing existing drugs.

**Table 2:** Investigator-associated phase II trials supported by the KOSMOS-Industry consortium, available as Tier 3A options in the KOSMOS-II trial

	**AL22-16 (BRISK)**	**AL22-27 (BROAD)**	**AL23-02 (METASIS)**	**AL23-12 (KRAUS)**	**AL23-17 (GAUSS)**
Clinical trial registration number	KCT0007840/ TBD	KCT0008405/ NCT05876806	KCT0008450/ NCT05882292	NCT05993455	TBD
Investigational products	Bevacizumab + Erlotinib	Dabrafenib + Trametinib	ABN401	Sotorasib + Panitumumab	Niraparib
Eligible GAs	LoF alterations in the genes regarding Kreb cycle *- IDH1* *- IDH2* *- SDHB* *- SDHA* *- SDHD* *- FH*	*BRAF* mutations < Class I > *BRAF* V600E/K/D/R/M (Excluding V600E/K melanoma, V600E non-small cell lung cancer and V600E colorectal cancer) < Class II > P367L/S, G464V/E, G469A/V/R/S, L485W, E586K, L597Q/R/S/V, T599TT/TS, T599I/K, K601E/N/T, Fusion of *BRAF* kinase domain, *BRAF* kinase duplication	- *MET* exon 14 skipping mutation - *MET* amplification ≥ 6 copies by NGS	*KRAS* G12C (excluding non-small cell lung cancer and colorectal cancer)	LoF alterations in genes related to homologous recombination repair (excluding ovarian and prostate cancer) Homologous recombination deficiency in whole-genome sequencing

*Abbreviations: TBD* to be determined, *LoF* loss of function, *GA* genetic alteration, *NGS* next-generation sequencing

### Study process

After a participant signs an informed consent, their clinical information is entered into the electronic case report form, which automatically sends the information towards the virtual cMTB platform, NAVIFY®. The participants’ NGS report is separately uploaded to NAVIFY®. Seven cMTB panels are organized, each comprised of three to four medical oncologists and at least one or more pathologists or bioinformaticians. The virtual cMTB meetings are held twice a week. To ensure the participants’ personal information protection, the clinical information on NAVIFY® is pseudo-anonymized, and all members of the cMTB panels sign confidentiality agreements.

Overall, cMTB provides the following information after discussing each case:

1. Actionable genomic alteration from the subject’s NGS report
2. Preferred recommendations: (Tier 1, Tier 2, or Tier 3) (Fig. 1)
3. Level of evidence per recommendation.

The level of evidence is assigned according to the Korean Precision Medicine Networking Group scale of clinical actionability of molecular targets (K-CAT) [12]; supporting references regarding the assigned K-CAT levels should also be provided. The cMTB can suggest a maximum of three treatment options in a hierarchy, and the investigator who submits the case can choose among the provided options.

**Fig. 2:**
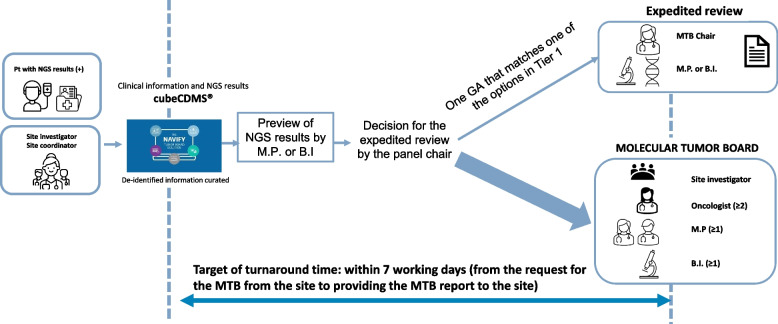
Central molecular tumor board workflow in the KOSMOS-II trial. Abbreviations: NGS, next-generation sequencing; M.P, molecular pathologists; B.I, bioinformaticians; GA, genetic alteration; Pt, patient

The cMTB process is illustrated in Fig. 2. The NGS report is reviewed and commented on by the panel pathologist and/or bioinformatician prior to the meeting. If a patient exhibit only one actionable GA that matches one of the treatment options of Tier 1, the case can be assigned to Tier 1 treatment through an expedited review by the panel chair (a medical oncologist), pathologist, and/or bioinformatician, without being fully reviewed by all cMTB members on an online forum. If a case is not recommended for expedited review, the medical oncologists on the panel search for clinical trials available in Korea that are relevant to the submitted case. This process involves checking the MFDS website (https://nedrug.mfds.go.kr/index), Korea Disease Control and Prevention Agency Clinical Research Information Service (https://cris.nih.go.kr/cris/index/index.do), and the Korean Cancer Study Group (KCSG) website (https://www.kcsg.org). Treatment options are determined after a discussion on the online forum regarding the interpretation of GAs and the available options obtained through the search.

The status of various clinical trials of MGT in multiple institutions is monitored and curated by the KOSMOS-II study team and regularly provided to the cMTB panel members. Online forums and educational workshops for cMTB are organized and supported by the Korean Society of Medical Oncology.

### Study assessments

Pretreatment evaluations require clinically appropriate radiographic studies to assess target or nontarget lesions for each participant, according to RECIST v1.1 criteria. For those who participate in Tier 1, tumor assessments are performed every 8 ± 2 weeks, according to RECIST v1.1 or iRECIST (for those who were treated with atezolizumab). For those who participate in Tier 3A trials (Table 2), tumor assessment is performed according to the protocol of each study, and the intervals between assessments range from 6 to 8 weeks.

Tier 1 serious adverse events are collected according to CTCAE v5.0. In Tier 3A studies, adverse events are identified and assessed according to each protocol.

### Translational projects and the master protocol

Several translational projects utilizing the KOSMOS-II trial are ongoing. The translational explorative objectives are as follows:

1. To correlate the response to immune checkpoint inhibitors with tumor mutational burden confirmed by local NGS testing.
2. To correlate the response to immune checkpoint inhibitors with the Lunit SCOPE IO, an artificial intelligence-powered spatial tumor-infiltrating lymphocyte analysis of digitized data from qualified scanned images of hematoxylin and eosin-stained slides. This analysis demonstrates its predictive role in various types of tumors, including non-small cell lung cancer [13].
3. To illustrate the genomic landscape of Korean patients with solid tumors through whole-genome sequencing (WGS) of participants who provided recently obtained tumor tissue (within 3 months from enrollment) and to explore the possibility of using WGS to identify the appropriate MGT.

### Establishment of a clinico-genomic database

**Fig. 3:**
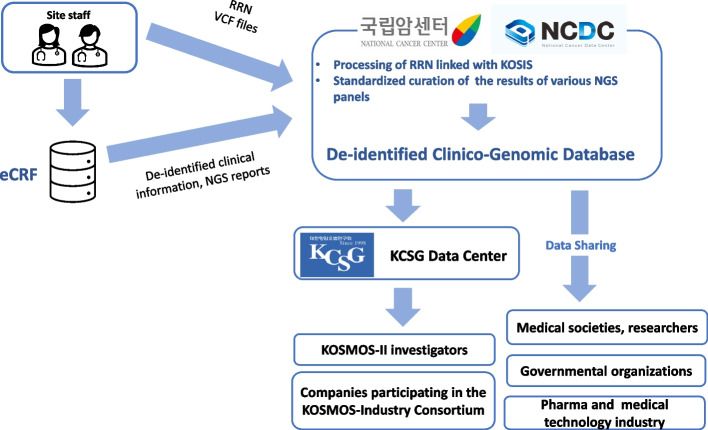
Representation of the flow of integration and distribution of clinico-genomic data in the KOSMOS-II trial. Abbreviations: eCRF, electronic case report form; RRN, resident registration number; VCF, variant call format; KOSIS, Korean Statistical Information Service; NGS, next-generation sequencing; KCSG, Korean Cancer Study Group

A specific collateral project of this study is the establishment of a nationwide CGDB in collaboration with the National Cancer Center of Korea (NCCK), designated the National Cancer Data Center (NCDC). The clinical characteristics of the participants, their genomic profiles, and outcomes of the MGT (Tier 1 and Tier 3A) are stored as anonymized codes and curated in the CGDB for further research and development. The resident registration numbers of the study participants are collected and transferred to the NCDC, where they are linked to the data by the Korean Statistical Information Service to update the survival status of the participants after the study ends. Efforts, such as collecting the metadata of each local NGS panel and transforming heterogeneous genomic variant call format (VCF) files into a standardized format, are ongoing to build the CGDB (Fig. 3).

KOSMOS-II investigators and companies participating in the KOSMOS Industry Consortium will have access to the CGDB, which will be transferred to the data center of the KCSG, the sponsor of this study. The KCSG has a partnership agreement with the NCCK to ensure and promote broad data sharing among academic societies, governmental organizations, and biopharmaceutical companies. The CGDB housed in the NCDC will be made available to the public 3 years after the completion of this study and distributed according to the pre-specified governance rules for the data.

### Statistical considerations

Given that MGT was available for 51.3% (99/193) of patients in the KOSMOS-I trial [14], it is expected that the MGT rate in this study will be approximately 50%. To estimate the proportion of patients with a 95% confidence interval of 0.065, a total of 950 patients need to be included. Considering a 5% dropout rate, the plan is to enroll 1,000 patients in this study.

Considering the CBRs or disease control rates in previous genomically guided basket trials have ranged from 20 to 50% [4, 15, 16], the expected CBR of Tier 1 patients is 30%. Assuming the null hypothesis that the CBR is 20% or less, as might be expected from studies on investigational products reported in the early 2000s [17], 221 patients are needed to demonstrate that the true CBR is 30%, with a type I error of 5% (2-sided) and 90% power. Considering a 20% dropout rate, we need to enroll 265 patients in Tier 1. In addition, we will enroll approximately 35 exceptional responders from KOSMOS-I trial, resulting in a total of 300 patients being enrolled in Tier 1 for this study. The scope of data collection according to the endpoints in each tier is presented in Table 3.

**Table 3:** Data collection scope in the KOSMOS-II Trial

	**Tier 1**	**Tier 2**	**Tier 3**	
			**Tier 3A**^**a**^	**Tier 3B**^**b**^
**MGT rate**^**c**^	O	O	O	O
**16w-CBR**^**c**^	O		O	
**ORR**	O		O	
**PFS**	O		O	
**DoT**	O		O	
**1y-OS**	O	O	O	O
**Safety**	O		O	

*Abbreviations: MGT* molecular profiling guided therapy, *16w-CBR* clinical benefit rate at 16 weeks, *ORR* overall response rate, *PFS* progression-free survival, *DoT* duration of treatment, *1y OS* overall survival at 1 year

^a^Clinical trials supported by KOSMOS industry consortium

^b^Clinical trials not related to KOSMOS-II framework

^c^Co-primary endpoint

### Current status

This study enrolled its first participant in September 2022 and 418 patients (42% of the projected number) as of October 2023. A total of 31 institutions across Korea participated in this trial, with 29 of the 31 institutions having started to enroll patients. The virtual cMTB held its 110th meeting in October 2023. The expected duration of this study is 3 years, including 2 years of enrollment and 1 year of follow-up.

## Discussion

This study is a type of MOT, a prospective observational trial that enrolls patients based on a precise molecular biomarker testing algorithm and incorporates interventional trials or real-world data (RWD). MOT is a new class of master clinical protocols proposed to bridge the gap between interventional trials and retrospective RWD in data collection for precision medicine [18].

MOT, such as the KOSMOS-II trial, can provide a common screening platform for various collateral studies. Identifying rare actionable GAs is challenging without comprehensive molecular testing in a large number of patients. The opportunity to offer patients innovative treatment options hinges on the seamless integration of the interpretation of molecular test results and drug-access programs, including clinical trials. The KOSMOS-II trial employed various NGS panels certified by healthcare authorities, such as MFDS, to efficiently screen participants and reduce the time and cost associated with testing and logistics.

In Korea, NGS for clinical diagnosis can only be performed by clinical laboratories certified according to the laboratory guidelines of MFDS. Most laboratories perform comprehensive genomic profiling, testing hundreds of genes, and follow the good laboratory standards of the Korean Society of Pathologists [8]. However, the heterogeneity of the panels in terms of sequencing methods, covered gene regions, and variant calling pipelines poses challenges in constructing a molecular matrix for MOT. To address these issues, the KOSMOS-II study team collects metadata for each submitted NGS panel, including the list and number of targeted genes, sequencer type, reference sequence, target capture kit, and variant-calling software. Even in the absence of a unified testing platform, this standardized curation of detected variants for each participant helps to provide reliable information about the actionability of the GAs. The Targeted Agent and Profiling Utilization Registry (TAPUR™) trial has already demonstrated that heterogeneous molecular testing methods can serve as platforms for precision medicine trials [3]. Our trial is anticipated to provide evidence supporting an efficient and pragmatic framework for nationwide screening in the realm of drug development and repositioning.

RWD is increasingly used to assess the impact of drugs in daily practice and to support the drug approval process, helping to address the limitations of clinical trial data. It can provide information about underserved populations or individuals with underlying diseases or organ dysfunction who are ineligible for clinical trials [19]. However, the accuracy of data, such as molecular annotations or clinical events (disease progression, recurrence, or adverse events from treatments), for cancer patients is often questioned in RWD because of the heterogeneity of testing methods and the result reporting, as well as non-standardized reports regarding efficacy and safety outcomes [18]. MOT can mitigate the disadvantages of RWD by prospectively collecting clinical data using a common case report form, while treatment and evaluation schedules are more flexible compared with those of traditional clinical trials [20]. In the KOSMOS-II trial, clinicopathologic information for all registered patients is being collected prospectively, and the response to MGT in Tier 1 and Tier 3A patients is being recorded according to the RECIST 1.1 criteria (also iRECIST for patients receiving immune checkpoint inhibitors).

Many precision medicine trials have shown that one or more potentially actionable GAs can be identified in approximately 30%–50% of patients, with approximately 10–20% of them being eligible for MGT [1, 2, 4, 6, 16, 21]. Some studies have demonstrated improved clinical outcomes with MGT compared with conventional therapy [2, 6]. The proportion of patients eligible for MGT can vary depending on the type of molecular profiling platform, the availability of MGT options, and the investigators’ treatment intentions. As demonstrated by our previous study, KOSMOS-I, we achieved a matching rate of 51.3% by implementing the cMTB combined with the TUID program, along with efficient communication for clinical trial enrollment among cMTB members and investigators. This rationale led us to hypothesize that the MGT rate is 50% in KOSMOS-II. The expected CBR in Tier 1 is 30%, which is a significant improvement for heavily treated and refractory populations. However, achieving desired efficacy levels may be challenging because in many cases, the genomic profiles for MGT are derived from the sequencing results of tumor samples obtained significantly earlier than the treatment, which may not accurately reflect the genomic profile at the time of MGT implementation. To address these concerns, we encourage investigators to submit NGS results from the most recently obtained tumor samples and to enroll patients in a WGS translational project using fresh tissue samples.

To the best of our knowledge, this is the first multicenter, nationwide precision medicine study that utilizes cMTB for all participants. While discussions on MTBs may positively affect treatment decisions [22], increase the likelihood of enrollment in clinical trials, and potentially improve clinical outcomes [23], the associated turnaround time and cost might prevent the adoption of MTB in clinical practice [24]. Additionally, MTBs have not been actively employed in many Korean institutions because of disparities in human resources, infrastructure for NGS interpretation, and clinical trial access [9]. The KOSMOS-II trial involves 31 institutions from all over South Korea, and 12 of them are located outside the Seoul metropolitan area. To facilitate cMTB in this large-scale study, we use a video-conferencing platform and clinicopathologic information curating software, NAVIFY®. Our study aims to measure the operational feasibility of a nationwide cMTB by assessing the turnaround time from the site’s request to the cMTB’s decision, and by examining the agreement rate between the cMTB’s recommendation and the actual treatment administered to patients. Furthermore, we aim to gauge the consensus among different panels on similar cases, particularly in assigning a level of clinical actionability to individual cases.

Through the KOSMOS-II trial, we also aim to develop a CGDB that includes participants’ clinical characteristics and genomic profiles by processing VCF files from various platforms, which will be linked to reliable survival data provided by national mortality statistics. Several collaborative efforts have been made to develop real-world CGDBs to assess the effects of genomic profiling and MGT on patient care [25, 26]. The CGDB for the KOSMOS-II study will contribute significantly to precision medicine by providing high-quality clinical data and accurate genomic information.

In conclusion, the KOSMOS-II trial is designed to test the hypothesis that cMTB-based MGT approach is both feasible and effective for treating refractory solid tumors on a nationwide scale. This MOT, incorporating RWD and IITs, is expected to provide an efficient platform for identifying new indications or biomarkers for existing drugs as well as investigational agents. In addition, the CGDB developed through the KOSMOS-II trial may contribute to collaborative efforts aimed at data-informed clinical decision-making.

## Data Availability

No datasets were generated or analysed during the current study.

## Abbreviations

NGS: Next-Generation Sequencing
MGT: Molecularly Guided Treatment
MTB: Molecular Tumor Board
cMTB: Central Molecular Tumor Board
TUID: Therapeutic Use of Investigational Drugs
MFDS: The Ministry of Food and Drug Safety
MOT: Master observational trial
IIT: Investigator-initiated trial
CGDB: Clinico-Genomic Database
GAs: Genetic Alterations
CBR: Clinical Benefit Rate
RECIST: Response Evaluation Criteria in Solid Tumors
CTCAE: Common Terminology Criteria for Adverse Events
HIRA: The Health Insurance Review and Assessment Service
K-CAT: Korean Precision Medicine Networking Group scale of clinical actionability of molecular target
KCSG: Korean Cancer Study Group
WGS: Whole genome sequencing
NCCK: National Cancer Center of Korea
NCDC: National Cancer Data Center
VCF: Variant Call Format
RWD: Real-World Data
TAPUR: Targeted Agent and Profiling Utilization Registry trial
